# The Effect of the Protein Synthesis Entropy Reduction on the Cell Size Regulation and Division Size of Unicellular Organisms

**DOI:** 10.3390/e24010094

**Published:** 2022-01-07

**Authors:** Mohammad Razavi, Seyed Majid Saberi Fathi, Jack Adam Tuszynski

**Affiliations:** 1 Department of Physics, Faculty of Science, Ferdowsi University of Mashhad, Mashhad 9177948974, Iran; sm.razavigovareshk@mail.um.ac.ir; 2 Department of Oncology, University of Alberta, Edmonton, AB T6G 1Z2, Canada; jack.tuszynski@gmail.com; 3 Department of Physics, University of Alberta, Edmonton, AB T6G 2E1, Canada; 4 Department of Mechanical and Aerospace Engineering, Politecnico di Torino, 10129 Torino, Italy

**Keywords:** protein synthesis, entropy reduction, free energy, cell size

## Abstract

The underlying mechanism determining the size of a particular cell is one of the fundamental unknowns in cell biology. Here, using a new approach that could be used for most of unicellular species, we show that the protein synthesis and cell size are interconnected biophysically and that protein synthesis may be the chief mechanism in establishing size limitations of unicellular organisms. This result is obtained based on the free energy balance equation of protein synthesis and the second law of thermodynamics. Our calculations show that protein synthesis involves a considerable amount of entropy reduction due to polymerization of amino acids depending on the cytoplasmic volume of the cell. The amount of entropy reduction will increase with cell growth and eventually makes the free energy variations of the protein synthesis positive (that is, forbidden thermodynamically). Within the limits of the second law of thermodynamics we propose a framework to estimate the optimal cell size at division.

## 1. Introduction

Entropy is an important concept first defined in thermodynamics and developed in statistical mechanics but also applied outside physics including the biological sciences, where it has been used to explain various problems ranging from intracellular considerations to the biosphere [1,2,3,4,5]. It provides a quantitative measure of biological energetics and enables assessment of the amount of disorder in processes taking place in living organisms and within their functional components [6]. Entropy-based analyses, which can help reduce time and cost, are also useful in developing computational drug discovery methods [7,8,9]. Entropy in the context of data related to biological systems is increasingly being applied in investigations associated with health and disease. For example, entropy has been used to demonstrate that cancer cells cannot decrease entropy to the same extent as healthy cells. Hence, entropy change can be used as a prognostic tool providing a measure of malignancy [3,10]. Cell death becomes evident in the destruction of structures and functions that cause continuous entropy production, which is consistent with the second law of thermodynamics that predicts a spontaneous tendency to increase entropy in closed systems [1].

During the process of protein synthesis, from the view of information theory, the entropy of amino acids is reduced compared to the entropy value for the corresponding DNA sequence that codes for this protein [1]. On the other hand, the more comprehensive and physical view of thermodynamics tells us to offset this entropy reduction; the process needs a large amount of energy provided by the hydrolysis of ATP, which in general leads to an overall increase of entropy in the biological system and its environment (to learn more about these views please refer to [11,12,13]). This process is performed in an irreversible manner as a direct result of the central dogma of molecular biology [14]. 

Thus far, the entropy reduction of protein synthesis has been calculated based on the difference in the information capacity of a gene (as the initial state), the resultant protein (as the final state) [1], and the obtained value of −1.16R (R is the gas constant) for entropy reduction of this process. In another study, this entropy reduction has been estimated based on the minimal free energy required for the string writing process of protein translation, taking a uniform bath of amino acids (or a pool of free-floating amino acids) to a specific protein [2]. Both of these studies have obtained the same order of entropy reduction rate. These authors have estimated that the total cost of the process is at least 120 kJ/mol (this is the standard Gibbs free energy $\Delta G^{\prime {}^{\circ}}$) or, equivalently, it is accompanied by the entropy increase of 48R, which is greater by two orders of magnitude than the corresponding entropy reduction in this process. Now the following questions arise:

1. From the viewpoint of the second law of thermodynamics, could protein be considered to be the final state of a given gene?
2. If the cells used a different alphabet for gene/mRNA and, more specifically, if they used a system based on more/fewer than four nucleic acids as codes for storing information on protein synthesis, would the change in the entropy of the amino acids be different from the current value?

In this study, these questions are addressed and relevant conclusions are drawn. In fact, the main part of entropy reduction, which is missing in prior studies [1,2], is due to the aggregation of amino acids from the whole cell to a tiny space of protein that dramatically shrinks the configuration part of the phase space of amino acids and the number of microstates of the system. This phase space reduction aspect, or compartmentalization, controls the structure formation in the cell [1]. In statistical thermodynamics, to properly define the phase space and specifically determine whether the elements follow Markovian or non-Markovian interaction types is the central issue. We show below that this entropy reduction is cell size-dependent, and related conclusions link this result to the long-standing open problem concerning how the specific size of individual cells and their size of division (maximum cell size) can be determined (and the underlying biophysics of this problem) [15,16,17]. More specifically, Ginsberg et al. addressed the problem by posing the following questions [18]:

1. Do animal cells have mechanisms to autonomously measure and adjust their individual sizes?
2. Does the presence of such mechanisms indicate that there is an optimal cell size for a particular cell’s function?

Cell size is an important physiological characteristic that regulates the scale of all biosynthetic processes. Although physiological studies have revealed that cells actively adjust their size, the governing rules underlying this regulation have remained unclear. Due to its vital role in cellular processes, many organisms actively control cell size by coupling growth and division [18,19,20], and maximum cell size is often restricted by environmental conditions [20].

Proteins constitute about 50% of the dry mass of a living cells and are the most abundant macromolecules in living cells present in their membranes, cytosol, cell organelles, and chromosomes. Therefore, recognizing the relevant variables of protein synthesis, especially the precise estimation of its entropy changes, would provide an insight into the underlying physico-chemical postulates of cell biology. We believe that to compare the rate of entropy reduction in protein synthesis with the total energy cost of the process, the real/physical entropy reduction should be considered. 

The structure of this article is as follows: The governing thermodynamics principles of protein synthesis are clarified in Section 2 by introducing the relevant free energy balance equation. In the next section, after answering the above questions, the entropy reduction in the process of protein synthesis is calculated and based on the second law of thermodynamics, suggesting a link to the problem of cell size control and optimization. Section 4 is devoted to the mutual relation between cell size (more precisely cytoplasmic volume) and the entropy reduction rate of protein synthesis. In Section 5, the validity of the results is shown in accordance with empirical evidence. The subsequent section explains how the second law of thermodynamics by the free energy balance equation of protein synthesis imposes an upper bound on the living cell’s size and forces cells to initiate the division process in order to avoid cell death.

## 2. Free Energy Balance Equation of Protein Synthesis

From the biophysical viewpoint, the most important matter in protein synthesis is the energy balance of this process, which in this case is determined by the Gibbs free energy equation:

(1) G=U+PV−TS

Based on the second law of thermodynamics, an open system such as a living cell (at a constant temperature T) can spontaneously drive a process if the total outcome of the process reduces the free energy of the system and environment [1,21]:

(2) $$\Delta G_{\mathrm{protein}\;\mathrm{synthesis}}=\Delta G_{\mathrm{system}}+\Delta G_{\mathrm{environment}}\leq 0$$

This condition on the free energy variations, governs the process of protein synthesis in which a peptide chain is formed (entropy reduction reaction) at the cost of the energy of ATP hydrolysis (entropy production reaction). Therefore, we have:

(3) $$\Delta G_{\mathrm{protein}\;\mathrm{synthesis}}=\left|U_{\mathrm{Peptide}\;\mathrm{Bond}}\right|-\left|U_{\mathrm{ATP}\;\mathrm{Hydrolysis}}\right|-T\Delta S_{\mathrm{Amino}\;\mathrm{Acids}}\leq 0$$

**Figure 1:**
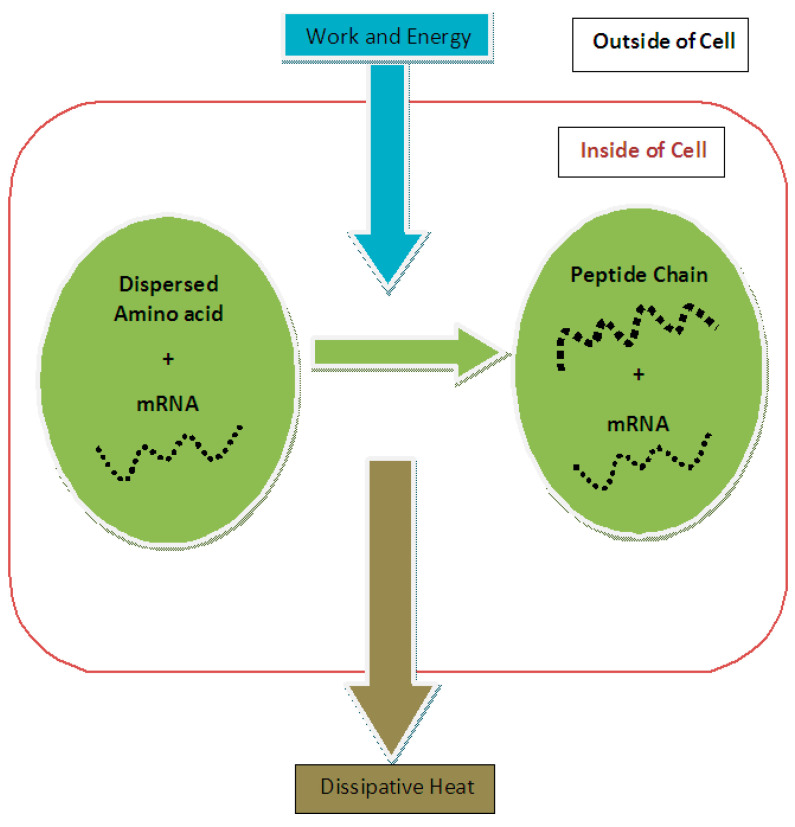
Schematic representation of protein synthesis in a biological cell viewed as a thermodynamic engine.

The first term on the right-hand side is the required free energy for the endoergic reaction of the peptide bond formation that is provided by the second term (i.e., the hydrolysis of ATP), which provides the required free energy or input energy for the execution of the entire process of protein synthesis (see Figure 1). Therefore, based on input energy and effective output we could determine the efficiency of protein synthesis:

(4) $$\eta =\frac{\left|U_{\mathrm{Peptide}\;\mathrm{Bond}}\right|+\left|T\Delta S_{\mathrm{Amino}\;\mathrm{Acids}}\right|}{\left|U_{\mathrm{ATP}\;\mathrm{Hydrolysis}}\right|}$$

The theoretical upper bound on η could be obtained from the Carnot efficiency ($\eta_{C}$) of a heat engine (this upper bound is accessible only in an infinitely slow engine operation) that works between two sources of a hot reservoir at a temperature $T_{H}$ and cold one at $T_{C}$:

(5) $$\eta_{C}=1-\frac{T_{C}}{T_{H}}$$

In [22], it has been stated that: “The efficiencies of molecular machines, which operate isothermally and are often driven by the chemical potentials of activated molecules, like ATP, are not meaningfully restricted by the Carnot limit”. It is worth mentioning that Jaynes properly noted that in a biochemical process, $T_{H}$ is the highest temperature to which the activated molecules could deliver heat [23]. Therefore, here the temperature of the cold reservoir is equal to the biological temperature (about 300 K) and $T_{H}$ is the temperature of the tiny hot spot produced by the hydrolysis of ATP. If we consider the released energy is concentrated in only N=2 degrees of freedom (one vibrational mode) and $k_{B}T/2$ for each degree, with the released energy of at least $E_{\mathrm{ATP}}=30\;\mathrm{kJ}/\mathrm{mol}$ we will have [23]:

(6) $$T_{H}=\frac{E_{\mathrm{ATP}}}{N_{a}k_{B}}=3610\;K$$

where $N_{a}$ is the Avogadro number and $k_{B}$ is the Boltzmann constant. This temperature range is confirmed by the green and blue light emission of bioluminescence spectra that in insects is provided by the hydrolysis of ATP [24]. Bioluminescence is dependent on the concentration of luciferin and the light emitted is proportional to the ATP concentration [25]. Therefore, the Carnot efficiency of ATP hydrolysis gives the upper bound of 0.916 for η. In normal conditions of a typical living cell, ATP hydrolysis releases the energy of about 50 kJ/mol and Carnot efficiency has the value of approximately 0.95. However, in practice similar heat engines working near this upper bound are hardly possible. Based on empirical evidence, the efficiency of ATP hydrolysis is not fixed and depends on the correlated biochemical reaction and other circumstances (e.g., pH, temperature, etc.).

The last term in Equation (3) is the free energy change associated with entropy reduction of amino acids due to their assembly process during protein synthesis. So far, this entropy reduction has been considered from an information theory perspective [1,2]. We reconsider and calculate this term thermodynamically and focus on its contribution to the energetics of protein synthesis. Entropy is a state function of the system and simply depends on the initial and final states of the system, so the entropy change of protein synthesis should be independent of its path. In other words, since entropy is a state function of the system, a change in the entropy of a system is entirely determined by its initial and final states [26,27]. Therefore, entropy changes (reduction) for amino acids due to protein synthesis should be independent of the intermediate stages, especially the information that is used for its synthesis (genetic information or mRNA). In fact, based on the protein alphabet alone, it is unclear as to which system of coding translates the necessary information for protein synthesis. The total entropy increase in protein synthesis can be estimated as the energy of four high-energy phosphate bonds for each peptide bond formed, such that each amino acid is polymerized with a cost of at least 120 kJ/mol, and this is the cost of the whole process, containing four stages [1,28], not just for the entropy change during translation of information from mRNA to a peptide chain. This information pertains only to the deviation from the random distribution of amino acids to their specific order in a peptide chain. In other words, genetic information simply deviates from randomness or an *a priori* equal probability of amino acids in the peptide chain. Hence, this probability distribution bias can also be viewed as an entropy reduction process. Based on the thermodynamics, we believe that the main factor of entropy reduction in protein synthesis is the assembly process of amino acids that condenses them from a dispersed state in a cell volume (initial state) to a localized distribution in a protein volume (final state). Therefore, in response to the first and second question, we should mention the initial state of a protein is not represented by a corresponding gene, rather it is the individual amino acids forming this protein that are dispersed in the cell volume before being brought together to form a peptide chain. Also, following the above description, when the initial and final states of amino acids are known, the entropy change due to the process of forming a protein does not depend on which kind of alphabet is used to synthesize it. The contribution of this entropy reduction to the free energy balance equation of protein synthesis in our model contains interesting results and could link this problem to the long-standing question of cell size control [16,17,18].

## 3. Calculation of Entropy Reduction

For the purpose of calculating the entropy change in protein synthesis, one should consider the initial and final states of the process. In the initial state, this subsystem is composed of a number of dispersed amino acid molecules in the cytoplasmic solution of the cell plus mRNA, which contains the necessary assembly information for producing a peptide chain from appropriate amino acids and in the correct order. In the final state of this subsystem, there is a peptide chain that is formed by the amino acids localized at one area of the cytoplasm plus unchanged mRNA (see Figure 1). 

During the process of protein synthesis, mRNA remains unchanged so it can be used again for another similar process [29]. Path independence of entropy as a “thermodynamic state function of the system” allows us to calculate the entropy change based on the spatial congregation of amino acids due to their assembly as the main factor in the associated entropy reduction. We believe that a justifiable choice for an estimate of this entropy reduction is a method based on the concept of physical entropy (algorithmic complexity) introduced by Zurek [11,12]. He defined the concept of physical entropy by the size of the shortest message specifying the microstate uniquely up to the assumed resolution. He further used the coarse-graining concept in the cell’s volume of the phase space such that only a particle could be present in a cell. Then, he defined the physical entropy of the system as the length of the shortest message or computer program for a universal Turing machine that produces the output in a binary string [11], so that the algorithmic complexity of this configuration is given by:

(7) $$S=k_{B}N\ln\frac{V}{N\Delta V}$$

This equation represents the configuration part of the Sackur-Tetrode equation for the entropy of the ideal gas. Its complete form is given by [11]:

(8) $$S=k_{B}N\left(\ln\frac{V}{N\Delta V}+\frac{D}{2}\ln\frac{mkT}{{\left(\Delta_{p}\right)}^{2}}\right)$$

Here, $k_{B}$ is the Boltzmann constant, T the absolute temperature, while $\Delta V={\left(\Delta x\right)}^{D}$ and $\Delta_{p}$ define the resolution in the configuration and momentum halves of the *D*-dimensional phase space. A simple derivation of the Sackur-Tetrode equation can be provided as follows. There are $Ω=e^{N}/N!$ different possibilities of distributing N indistinguishable particles of gas among the available $e\approx \left(V/\Delta V\right){\left(\sqrt{mkT}/\Delta_{P}\right)}^{D}$ cells in the phase space. To stipulate a specific configuration is to assign its “address” among the Ω possibilities. The usual value of the address numeral is then ~Ω. Thus, its binary specification necessitates $\sim \log_{2}Ω$ bits. The Sackur-Tetrode equation follows. 

It is worth mentioning that here we refer to the concept of physical entropy just to show that the ideal gas is a reasonably good approximation to calculate the volume reduction entropy of amino acids without using it as a computational method. It should be stated that due to the temperature being kept constant during protein synthesis, the second term of the above equation, which only depends on temperature, remains unchanged. The change in entropy due to the change in the spatial aggregation of amino acids in the volume of a cell, $V_{1}$, in the beginning of the process when amino acid molecules are randomly dispersed in the cell, to the volume of a typical protein $V_{2}$, when the amino acids are incorporated in it, leads to:

(9) $$\Delta S_{\mathrm{Amino}\;\mathrm{Acids}}=k_{B}N_{a}ln\left(\frac{V_{2}}{V_{1}}\right)=Rln\left(\frac{V_{2}}{V_{1}}\right)$$

which is the same as the relation for the entropy change during an adiabatic contraction of a number of ideal gas molecules from volume $V_{1}$ to $V_{2}$ [30]. Moreover, this equation is the same as the one used for the calculation of the entropy change when dissolving a solid in a liquid [30]. However, dissolving is an irreversible process that leads to an increase in entropy. On the other hand, the amino acid contraction is exactly an opposite development that is an assembly (akin to crystallization) process. Therefore, they differ by a negative sign. For an elementary estimation, it is sufficient to assume that the initial volume (typical cell volume) is on the order of ${\left(\mu m\right)}^{3}$ while the final volume is on the order of ${\left(\mathrm{nm}\right)}^{3}$ (protein volume); thus, it is possible that the entropy change due to amino acid assembly is approximately −20R. This preliminary calculation modifies the order of the prior estimation obtained in [1]. In Ref. [1], it has been calculated that the entropy reduction due to protein synthesis is −1.16R and, similar to a refrigerator, the amount of energy and work that is consumed during this process leads to an increase of entropy up to $\Delta S_{environment}=48R$ [1]. 

Treating amino acids as an ideal gas gives an entropy reduction that, at least, gives a reasonable lower bound on the energetic cost to protein synthesis. This is due to the fact that the volume reduction entropy is somewhat independent of the physical parameters of the system and it is due to the significant shrinkage of the accessible volume of the phase space that is in fact the main part of entropy reduction of protein synthesis. Later, for the real conditions of the maximum cell size estimation of *E. coli* (Section 6), the Van der Waals fluid approach to compute the volume entropy reduction of amino acids is applied. It has the form of:

(10) $$S_{VW}=-a{\left(\frac{N}{V}\right)}^{2}V-NRT\ln\left(\frac{V-Nb}{V}\right)$$

Here constant a is a measure of the attractive forces between molecules and b is proportional to the molecules dimension [31].

As previously mentioned, some related efforts to calculate the entropy change of protein synthesis and its contribution to biophysical considerations can be found in the literature [1,2,4]. As an example, in [4], with the aim of estimating the entropy change due to bacterial cell division for *E. coli*, it was shown that entropy reduction of protein synthesis in comparison to entropy reduction due to bacterial cell division is relatively small. In light of our results, it is worth mentioning that the above statement is applicable to cells in the micrometer range since the rate of entropy reduction of protein synthesis is not fixed and should be considered as an increment function of the cell size.

As a further modification of Equation (9), if we consider unequal frequency for amino acid distribution in a living cell, which happens under real conditions (the effect of mRNA information), another term for entropy reduction, which comes from the Shannon information formula, should be added to obtain a more accurate estimate:

(11) $$\Delta S=N\left(-k_{B}\overset{20}{\underset{i=1}{\sum }}p_{i}\ln p_{i}-k_{B}\ln20\right)=Nk_{B}\left(2.8975-2.9957\right)=-0.0982R\;$$

in which $p_{i}$s are the probabilities of amino acid frequencies and N is the number of amino acids in a natural peptide. The frequency of amino acids could be found from either the Swiss-Prot or GenBank database [1,5]. However, these frequencies will change in different cells, the amount of related entropy reduction is negligible in comparison to the dominant value of −20R that is obtained in Equation (9), therefore we omit this term in next calculations.

Based on this result, it seems somewhat irrelevant to calculate entropy reduction of protein synthesis based on the difference between the final (protein) entropy and the initial (gene or mRNA) entropy as calculated in [1,5] since protein is not the final state of mRNA. Admittedly, Ref. [1] focused on the issue of entropy reduction and the balance of free energy provided by the ATP-consuming machinery of transcription and translation and not on a detailed calculation of entropic contributions. However, to properly address the latter issues, as shown here in Figure 1, a peptide chain representing a protein is the final state of dispersed amino acids and the entropy reduction of protein synthesis should be calculated based on these two states. Compared to references [1,2], in which the authors consider this problem to be based on information theory, the basic difference presented in our approach is that we discuss it in the context of thermodynamics and physical entropy. In this kind of problem, where one is faced with entropy reduction at the cost of a definite amount of energy and work, a thermodynamically-based approach seems fundamental. According to Zurek, a complete view into biological systems and their evolution is a combined physico-computational insight [11]. 

In a nutshell, from the dependence of the volume-reduction entropy on the cell volume it is evident that there is a mutual relationship between the cell size and the free energy balance equation of protein synthesis (Equation (3)). Cell size could affect the last two terms of the Equation (3) due to the dependence of entropy reduction of amino acids on it and since the high rate of ATP concentration could not continue with the increase of the cell size and they are negatively correlated (as a result of proportionality of the metabolic rate to the cellular surface-to-volume ratio) that leads to a decrease of accessible free energy of ATP hydrolysis [32]. Since we have thermodynamic limitations on the efficiency, this mutual relationship could not continue arbitrarily and will ultimately limit cell size. We next describe in detail how these features affect the cell’s operational efficiency and its dependence on the cell’s size.

## 4. Cell Size–Entropic Force Interplay

The results of the previous section show a significant impact of the entropic force on the cell size and the efficiency of the cellular machinery’s operation through protein synthesis. Considering Equations (9) and (10), it is obvious that the rate of entropy reduction in protein synthesis is not fixed and it depends on the cell volume, which contains the necessary amino acid molecules required for protein assembly. As shown above, $V_{2}$ is the volume of the peptide chain or protein; thus, it is fixed for a typical protein. However, $V_{1}$ represents the volume of the cytoplasm containing amino acids and the whole space of the cell except the nucleus; therefore, it is not fixed. Thus, the rate of entropy reduction should be an increment function of the cell size. It means that the rate of entropy reduction of a typical protein synthesis increases with the cell’s growth. Thus, these cells could generate more negentropy and increase their Gibbs free energy in the form of chemical thermodynamic potential due to the relation of ΔG=−TΔS. That is why the larger cells could operate more efficiently than smaller ones in the process of protein synthesis (i.e., they are more powerful and could save more energy per volume (see next section)). This could be interpreted as the biophysical reason of hypertrophy phenomena. Increasing muscle tissue volume and hence strength would be an example of this effect whereby, instead of their number, the volume of the individual muscle cell increases [33]. 

On the other hand, as a typical cell grows, regulating and providing the minimum required metabolic rate is faced progressively with the problem of the cellular surface-to-volume ratio [34,35]. Since the production rate of ATP is proportional to the nutrient that is received by the cells’ surface and ATP consumption rate is proportional to the cells’ volume, hence larger cells cannot maintain the high concentration rate of ATP to the same degree as smaller ones. Accordingly, the released free energy of ATP hydrolysis decreases since it depends on the concentration of the reactants and products of the reaction according to the following formula:

(12) $$\Delta G=\Delta G^{\prime {}^{\circ}}+RTlnQ_{r}$$

Here $Q_{r}$ is the reaction quotient that measures the relative amounts of products and reactants present during a reaction [28]. In our case, ATP hydrolysis predominantly produces ADP and phosphate ($P_{i}$), therefore:

(13) $$Q_{r}=\frac{\left[\mathrm{ADP}\right]\left[P_{i}\right]}{\left[\mathrm{ATP}\right]}$$

In living cells there are skillfully-organized mechanisms that keep ATP concentration levels high, so that $Q_{r}\ll 1$. This low value of $Q_{r}$ leads to an increase of available energy due to ATP hydrolysis (e.g., in *E. coli* it is about 55 kJ/mol [36]) which could be even higher. For example, it is estimated that the average value of ADP concentration for a muscle cell at rest is 25 μM, which leads to real free energy of ATP hydrolysis of 64 kJ/mol [28]. Therefore, as cells become larger, the concentration of ATP and the released free energy of its hydrolysis decreases, which exerts thermodynamic pressure on cellular growth. We believe that the free energy balance equation of protein synthesis (Equation (3)) is one of the most important equations determining the cell size and presumably contains the underlying biophysics of cell size control. As will be shown below, this equation can be used to predict maximum cell size or the size of a typical cell at division (see Section 6).

## 5. Empirical Evidence

While this result may appear counterintuitive, some supportive empirical evidence exists for its correctness. Based on an empirical formula, Kempes et al. have shown that the smallest cells are over an order of magnitude less energetically efficient than the largest cells in terms of their performance when adding an amino acid to the protein chain [2]. Moreover, they found that the fraction of translation for repair of damaged protein rapidly decreases with increasing cell volume, which is equivalent to a higher efficiency of larger cells. Therefore, based on the fact that an increasing efficiency of protein synthesis is paralleled by cell size increase, we expect that in a typical unicellular species, the process of natural selection would evolve in the direction of larger cells. This rational inference was previously validated in a long-term evolution experiment (LTEE) by Lenski et al. [37,38] who cultivated *E. coli* populations for more than 60,000 generations in a simple glucose medium. They found that increasing cell size coincides with an improvement in the fitness and faster growth rates [39], which was also confirmed by Gallet et al., but the reason why such a relation exists has remained elusive [40]. Here, for the first time we introduce a physical/thermodynamical explanation of this relationship. Also, our result supports the “internal diffusion-constraint” (IDC) hypothesis for the evolution of cell size [40]. Based on IDC there is a mutual effect between cell volume and metabolite concentration such that a reduction in the intracellular molecular traffic time would lead to a higher metabolism by increasing cytoplasmic cell volume. IDC hypothesis postulates that to better exploit and deplete glucose, natural selection would favor larger cells, which allows them to absorb more effectors than smaller cells can [40]. Moreover, in another work of Kempes [41], it has been shown that the evolution from unicellular prokaryotes to eukaryotes is correlated with changes in physiology and metabolic scaling in a manner that enables them to allocate an increasing portion of total metabolism to maintenance (instead of biosynthesis) with an increasing cell size.

## 6. Cell Growth Limitation Imposed by Carnot Efficiency

### 6.1. The Relation the Surface Area to Volume Ratio and Atp Concentration

The growth of an organism, whether multi- or unicellular, is a direct function of metabolic pathways which contain biochemical processes and biophysical conditions [42,43,44,45,46,47]. Also, the basic processes of cell physiology, such as material fluxes across membranes, are by their nature dependent on cell size. One of the most important growth factors that affects and limits the cell size is the ratio of the cellular surface area to its volume (SA/V). As a result, changes in cell volume and surface area have profound effects on metabolic fluxes, biosynthetic capacity, and nutrient exchange [35,48,49,50]. Generally, as the surface of cells is proportional to the square of length or diameter ($D^{2}$) and their volume is proportional to its third power, $D^{3}$, $SA/V\propto D^{-1}$ and since cells receive nutrient on the surface and consume it relative to their volume, it is reasonable to assume that in the growth phase, accessible nutrient of cells decreases with $D^{-1}$. Lack of nutrient leads to decreased ATP concentration, which affects the released free energy of its hydrolysis (see Equation (12)). Therefore, excess cell volume leads to cell death due to lack of nutrient and at a certain point in time of the growth phase, cells initiate division processes. Inhibition of cell division or more generally cell cycle progression leads to cell sizes that are substantially larger than cell size at normal and unperturbed division. Neurohr et al. [51] show that growing budding yeast and primary mammalian cells beyond a certain size causes cytoplasm dilution, contributes to aging and disrupts gene induction, cell-cycle progression, and cell signaling. These excessively large cells are not able to scale nucleic acid and protein biosynthesis in accordance with the cell volume increase [51].

On the other hand, as mentioned in previous sections from LTEE experiments and other studies, it is obvious that larger cells benefit from a relatively better efficiency than smaller ones and this is the choice dictated by natural selection. Hence, maximally increasing cell size is an advantageous tendency. Nonetheless, based on these data one could not define and explain the maximum size of a typical cell at division and justify it mathematically. Cell size at division is not fixed for different cells and even for a certain kind of a cell it depends on many factors such as nutrient quality, temperature, pH, and many environmental conditions, most of which affect ATP concentration [18,35,52]. In cells there are certain enzymes that sense ATP concentration and can contribute to signaling cell division initiation such as adenylate kinase (AdK) and its subfamily (see concluding remarks). In general, the main energy source for cellular metabolism is glucose, which is catabolized in the three subsequent processes (glycolysis, tricarboxylic acid cycle (TCA or Krebs cycle), and finally oxidative phosphorylation) to produce ATP [25]. The sum of all reactions for combustion of glucose is:

(14) $$\mathrm{glucose}\;+38\;\mathrm{ADP}\;+38\;\mathrm{Pi}\;+6{\;O}_{2}\;\to 6{\;\mathrm{CO}}_{2}+38\;\mathrm{ATP}\;+44{\;H}_{2}O$$

Moreover, in the reverse reaction of ATP hydrolysis to ADP and phosphate, the required free energy of biosynthesis processes is provided. One of factors that is needed to estimate the maximum size of a typical cell is the variations of the effective parameters on the reaction quotient of ATP hydrolysis $Q_{r}$ (see Equation (13)), with cell growth. Since the rate of glucose absorption is proportional to the cell’s surface area (SA), ATP production is related to SA and since ATP consumption rate (or ADP and Pi production rate) is proportional to the cell’s volume (V) during cell growth, $Q_{r}$ would change as a function of V/SA. Therefore, for the free energy variations of ATP hydrolysis we have:

(15) $$\Delta G=\Delta G^{\prime {}^{\circ}}+RTlnQ_{r}=\Delta G^{\prime {}^{\circ}}+RTln\left[f\left(V/SA\right)\right]$$

Although the exact form of f(V/SA) should be obtained from applying nonequilibrium thermodynamics [31] to the coupled reactions of ATP cycle, here the linear functional dependence as the first order approximation is considered:

(16) $$Q_{r}=C\left(V/SA\right)$$

where C is a positive parameter with dimension of $L^{-1}$ and defined based on the preliminary conditions of free energy of ATP hydrolysis and its concentration that during cell growth would evolve with the first Fick law. It relates the diffusion flux, j, to the gradient of the concentration, C, and states the direction of flux is toward the region of lower concentration:

(17) $$j=-D\frac{dC}{dx}$$

Here, D is the diffusion coefficient.

### 6.2. Methods and Results

In the following, a framework to compute and estimate the maximum size of a cell based on the empirical data is introduced. Going beyond the estimated maximum cell size is disadvantageous and the cell is faced with a thermodynamic penalty that stops most biochemical processes. We apply this framework for the estimation of the maximum size of Escherichia coli at the certain experimental conditions and the related data that is previously established by others [53,54,55]. We approximate the shape of *E. coli* by a capsule with length *h* and radius *r* for its caps and use the methods and data of length and diameter of the *E. coli* from Ref. [53] and the data of intracellular ATP concentration from Ref. [55]. With the help of statistical optimization methods, data is rearranged to obtain the best order for the next steps. However, designing an experiment with the goal of the finding of the free energy variations of ATP hydrolysis with the cell growth improves the estimation of the cell size at division.

To compute the concentration variations of ATP with increasing cell size the first Fick law (Equation (17)) is applied for the diffusion in a capsule-like vessel of *E. coli* plus the assumption that during growth, the fraction of surface area of the cell to its volume is almost constant [54]. Variations of the energy consumption by the peptide bond energy of formation is computed based on the classic allometric scaling relationship relating metabolic rate (B) to body mass (M) [56]:

(18) B=B0M34

(With $B_{0}$ being a normalization coefficient), was formulated first for mammals and birds by Kleiber in the 1930s [57], but now it is validated for the wide range of organism. Another term is related to the volume-reduction entropy (Equation (10)) that is computed based on the Van der Waals fluid approximation. For the parameters of the Van der Waals equation, we use $a=9/8RTV_{2}$ for attractive intermolecular potentials and chose $Nb=V_{2}/3$ [31] with $V_{2}=500\;{\left(\mathrm{nm}\right)}^{3}$ as the final volume of a vessel containing an average protein and given that the average protein length is 325 amino acids [2] plus the average volume for each amino acid is $141\;{Å}^{3}$ [58].

**Figure 2:**
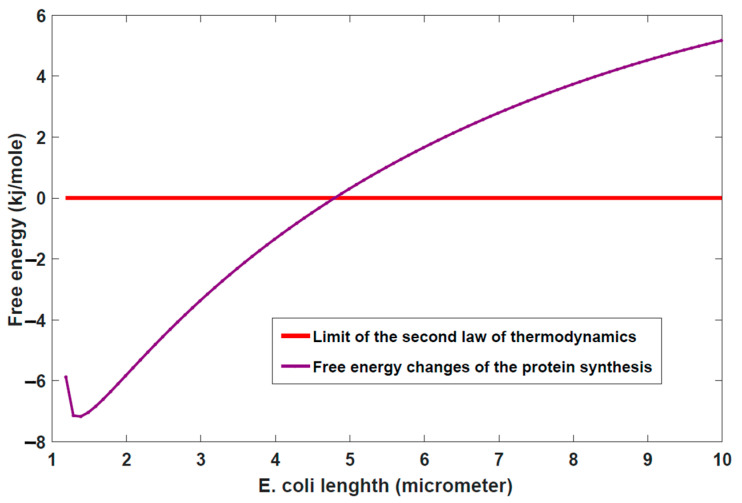
The free energy of protein synthesis becomes positive at cell length of about 4.8 μm which is disfavored by the second law of thermodinamics.

**Figure 3:**
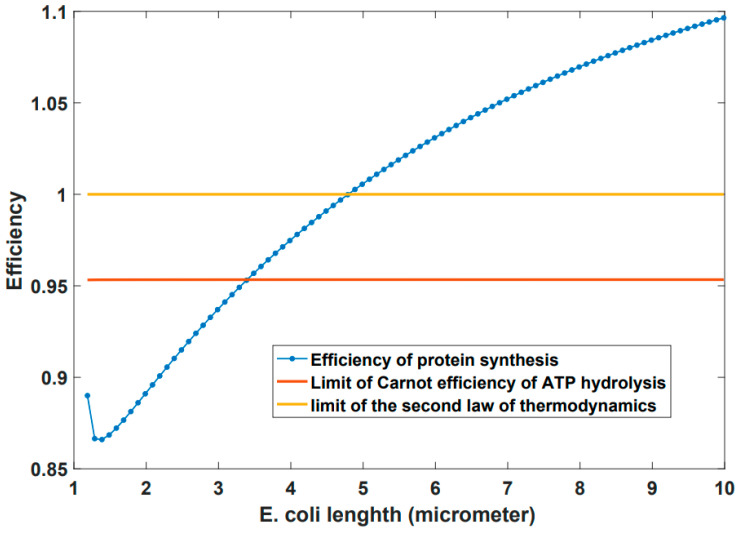
Before the limit imposed by the second law the process of protein synthesis is halted at 3.4 μm due to the Carnot efficiency limitation of ATP hydrolysis.

It should be noted that all of the terms of the energetics of protein synthesis (Equation (3)) are volume/cell size dependent, therefore a strong correlation between cell size and the process of protein synthesis does exist. Based on the above algorithm it is possible to find or predict the maximum cell size and cell size at division. As shown in the Figure 2 in *E. coli* the free energy changes of the process of protein synthesis become positive at length about 4.8 μm which makes the process impossible by the second law of thermodynamics but before this bond the process is halted at 3.4 μm due to the Carnot efficiency bond of ATP hydrolysis (Figure 3). This result is in good agreement with experimental measurements of maximum *E. coli* length of Ref. [54]. 

From these illustrations and the empirical evidence of the tendency of cells to become larger and more efficient (see Section 5) we believe that for each type of cell an optimal cell size range should exist, which is determined biophysically by the free energy balance equation of protein synthesis and its condition that ΔG≤0 which is due the second law of thermodynamics. It should be noted that energy production consideration may have additional effects on the size limitations as they enter into the thermodynamics and free energy balance equation of protein synthesis [59]. In [16] Taheri-Araghi and et al. with precise experiments showed how the size of *E. coli* could change based on environmental conditions, especially the quality of nutrients. 

### 6.3. Theoretical Upper Bound Estimates of Cell Size

**Figure 4:**
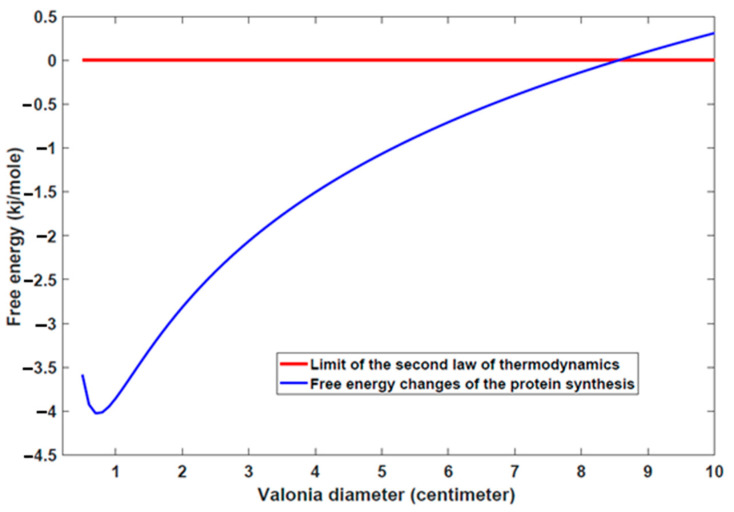
Maximum cell size that a giant single-celled like valonia could reach.

One of the most interesting points or questions that may come to mind is about the maximum cell size that could arise in Nature and what should be the biophysical properties of such a cell. It seems that the supposed cell should benefit from the high levels of ATP concentration and the related hydrolysis free energy. Reaching high levels of ATP concentration needs more nutrient which is harder for larger cells due to the small ratio of the cellular surface area to its volume (SA/V), therefore it is only possible by lowering the rate of energy consumption dramatically. Based on these explanations with the highest levels of ATP hydrolysis that have been reported in experiments (see Section 4 and [28,36]) a rough estimation of the upper bound of a living cell with a spherical shape approximation is considered. Our model shows that the diameter of the hypothetical cell could not be more than the order of centimeters, specifically 8 to 10 cm (see Figure 4.). Also, it should be noted that for different shapes this estimation could change. 

It seems that a giant single-celled organism called *Valonia ventricosa* would be an interesting example of this situation that has a spherical shape with a diameter that ranges typically from 1 to 5 cm [60]. As a plant Valonia grows and proliferates much slower than a typical bacterium such as *E. coli*. Taking advantage of several cytoplasmic domains that are interconnected by cytoplasmic bridges is the most important factor that helps Valonia to growing to this size. In this way, it seems that Valonia reduces the effective cytoplasmic cell volume used in entropy reduction of protein synthesis. Similar to Valonia in most of the species we suffer from the lack of enough experimental data specially the free energy of ATP hydrolysis and correlated data of cell size to test our model. We hope that this work would result in the design of targeted experiments to validate and improve the results obtained here.

## 7. Relation to Abiogenesis

The most obvious characteristic feature of life is information storage, which also leads to entropy reduction. The life cycle causes this entropy reduction due to the production of ordered structures in the form of macromolecules, such as proteins or by compartmentalization of matter (e.g., ions). Within the literature it has been found that any living system needs a physical boundary in order to separate life processes from non-living matter and the associated physical processes that evolve in the direction of ever-increasing entropy [61,62]. 

Furthermore, based on the results, which are obtained here, it is obvious that the process of macromolecule synthesis, such as proteins, should occur in a confined volume, which was indicated by $V_{1}$ in Equation (9), and the associated free energy balance equation leads to a size limitation within a submillimeter range in most species and ultimately could not be more than the order of centimeters in special cases [34]. Therefore, to start the process of macromolecule synthesis, volume confinement (membrane) is necessary to exist prior to protein and DNA formation. In other words, based on our calculations, the life cycle is not allowed to be initiated in an unrestricted-volume environment due to the second law of thermodynamics limitations. Accordingly, our results strongly support the hypothesis that the first self-replicating object should be lipid-like matter forming an enclosed membrane [63]. The gist of the idea proposed here is that the lipid-like amphiphilic molecular composition is the key precursor for information storage. Afterward, evolution may lead to the emergence of macromolecules such as protein or DNA that have the ability to store and even process information.

## 8. Concluding Remarks

What determines cell size? This question is one of the most important issues in mainstream cell biology [34,35]. Here, we attempted to find a biophysical answer, accounting for the second law of thermodynamics. This fundamental physical law is applied to the free energy balance equation for protein synthesis. By introducing the term of the volume-reduction entropy of amino acids in protein synthesis and its dependence on the whole cytoplasmic volume of the cell, we have illustrated that a reasonable upper limit on the size of each cell should exist that eventually forces the cell to divide. This upper limit on the cell size or volume also depends on the released free energy of ATP hydrolysis, which varies by ATP concentration. The framework introduced in this study provides a link between the macro-parameter of cell size and the micro-parameters of entropy reduction of protein synthesis and ATP concentration. The latter aspect is very significant since proteins constitute more than 50% of the dry mass of a living cell. Subsequently, it is strongly suggested that for integrated models of cell growth and division to fully understand multicellular growth and development, it is necessary to take the biophysical arguments into account [18,35]. One of the most important consequences of this result is that larger cells operate more efficiently energetically than smaller ones in the view of the protein synthesis, which is the most common and necessary process taking place in living cells [2]. In addition, it is expected that in most types of cells such as muscle, neuron, kidney, etc. and unicellular organisms, larger cells should have a higher operational efficiency than smaller ones, which is supported by ample empirical evidence [18,33,64,65,66]. 

Finally, it is worth addressing the following questions; based on this study what are the mechanisms that autonomously control and adjust individual cell size? And how does a cell determine the free energy changes during protein synthesis or in other words, how does an individual cell decide to divide before the second law of thermodynamics becomes violated in the process of protein synthesis (ΔG≥0)? Among the various possible answers, one seems more interesting: it is adenylate kinase (AdK) and its subfamily of phosphotransferase enzymes (AK1-AK7) that regulates the concentration of free adenylate nucleotides within the cell by catalyzing the conversion of ATP and AMP into two ADP molecules ($\mathrm{MgATP}+\mathrm{AMP}⇌2\mathrm{ADP}+{\mathrm{Mg}}^{2+}$). AdK plays an important role in cellular energy homeostasis by constantly monitoring phosphate nucleotide levels inside the cell [67]. At the conditions of low ATP concentration, they intervene to reduce ADP concentration by producing one molecule of ATP and AMP from two molecules of ADP through reaction of 2ADP↔ATP+AMP [68]. Therefore, AdK could affect Equation (13) in the direction of increasing free energy of ATP hydrolysis. Another function of AdK could be to signal the initiation of cell division since the free energy released due to ATP hydrolysis is no longer sufficient for biosynthesis processes. There are many reports providing empirical evidence confirming this claim. Some examples found indicate that recombinantly expressed AK2 in *E. coli* promotes cell growth and viability [69]. This could also be achieved by AK4 in some animal species, although it is enzymatically inactive and retains nucleotide-binding capability, it interacts with the mitochondrial ADP/ATP translocator and performs stress responsive protein function by promoting cell survival and proliferation [70]. In a recent study, it has been discovered that Arabidopsis AK6 impacts ribosome abundance, cell production and thereby root growth [71]. Therefore, these phosphotransferase enzymes probably are the main puzzle piece in the cell energetics and metabolic signaling networks having the ability to “measure” free energy balance equation of protein synthesis. 

The proposed model is still in a preliminary stage and could be further developed to obtain more realistic results especially since cell growth proceeds in multiple phases, each of which affects ATP concentration differently. Moreover, the computed volume reduction entropy of amino acids could be modified by numerical methods of entropy calculation in solutions. These modifications could help us in finding a precise value of ATP hydrolysis efficiency in protein synthesis. A detailed analysis of proper physico-chemical conditions that allow giant cells to reach their thermodynamic limit and deviate dramatically from normal range of cell size (submillimeter range) [34,35] should be a subject for future studies.

## Data Availability

Not applicable.
